# Nucleotide excision repair removes thymidine analog 5-ethynyl-2′-deoxyuridine from the mammalian genome

**DOI:** 10.1073/pnas.2210176119

**Published:** 2022-08-22

**Authors:** Li Wang, Xuemei Cao, Yanyan Yang, Cansu Kose, Hiroaki Kawara, Laura A. Lindsey-Boltz, Christopher P. Selby, Aziz Sancar

**Affiliations:** ^a^Department of Biochemistry and Biophysics, University of North Carolina School of Medicine, Chapel Hill, NC 27599

**Keywords:** excision repair, 5-ethynyl-2′-deoxyuridine, XR-seq, brain cancer

## Abstract

Nucleotide excision repair is the principal mechanism for removing bulky DNA adducts from the mammalian genome, including those induced by environmental carcinogens such as UV radiation, and anticancer drugs such as cisplatin. Surprisingly, we found that the widely used thymidine analog EdU is a substrate for excision repair when incorporated into the DNA of replicating cells. A number of thymidine analogs were tested, and only EdU was a substrate for excision repair. EdU excision was absent in repair-deficient cells, and in vitro, DNA duplexes bearing EdU were also substrates for excision by mammalian cell-free extracts. We used the excision repair sequencing (XR-seq) method to map EdU repair in the human genome at single-nucleotide resolution and observed that EdU was excised throughout the genome and was subject to transcription-coupled repair as evidenced by higher repair rates in the transcribed strand (TS) relative to the nontranscribed strand (NTS) in transcriptionally active genes. These properties of EdU, combined with its cellular toxicity and ability to cross the blood–brain barrier, make it a potential candidate for treating cancers of the brain, a tissue that typically demonstrates limited replication in adults.

## Significance

We discovered that the thymidine analog EdU, which is widely used in the analysis of DNA replication, DNA repair, and cell proliferation, is processed as “damage” in the human genome by the nucleotide excision repair system. EdU is unique in inducing DNA strand break and cell death of transformed cell lines. Our finding that EdU in DNA is processed in human cells as damage by nucleotide excision repair raises the possibility that such reaction causes a futile cycle of excision and reincorporation into the repair patch, leading to eventual cell death. Such a futile cycle leading to apoptosis makes EdU a potential candidate for the treatment of glioblastomas without serious side effects on postmitotic normal neural cells of the brain.

**Fig. 1.**
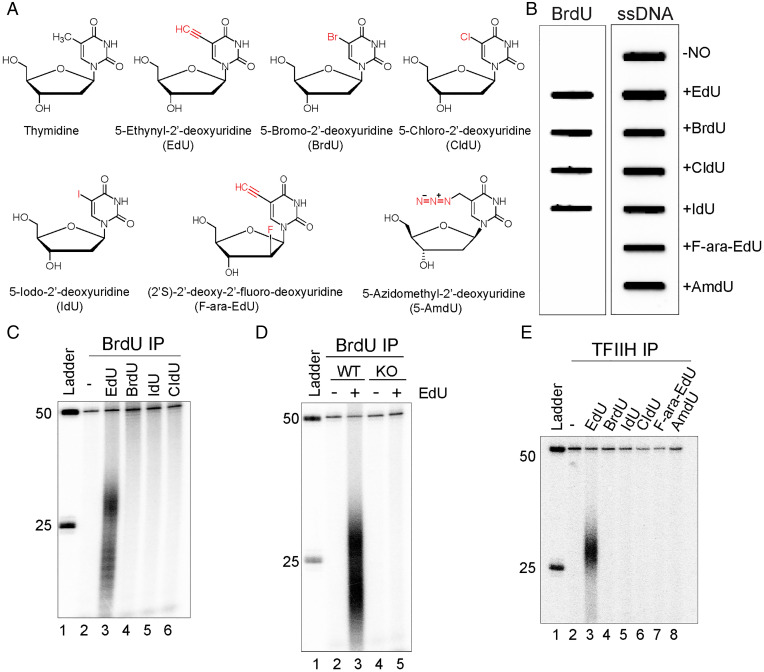
Incorporation of thymidine analogs into the human genome and removal of EdU by nucleotide excision repair. (*A*) Chemical structure of thymidine analogs used in this study. (*B*) Slot blot assay to test the reactivity of thymidine analogs with anti-BrdU antibody. HeLa cells were cultured either in regular medium or medium containing 10 μM thymidine analog (EdU, BrdU, CldU, IdU, F-ara-EdU, or AmdU) for 24 h. Genomic DNA was isolated, loaded onto a nitrocellulose membrane, and incubated with anti-BrdU or anti-ssDNA antibody. Anti-BrdU antibody recognizes BrdU, EdU, CldU, and IdU labeled genomic DNA. (*C*) Excision assay with anti-BrdU antibody immunoprecipitation. HeLa cells were treated with 10 μM thymidine analogs: EdU, BrdU, IdU, or CIdU for 24 h, then low-molecular-weight DNA was extracted by the Hirt procedure and immunoprecipitated with anti-BrdU antibodies. Purified, excised oligonucleotides were radiolabeled at the 3′ end together with a 50-mer as a spike-in internal control and separated on a DNA-sequencing gel along with a DNA ladder. (*D*) Removal of EdU requires excision repair. Human NHF1 wild-type and *XPA*^−/−^*XPC*^−/−^*CSB*^−/−^ mutant cells (knockout [KO]) were treated with or without 10 μM EdU for 24 h. Then, cells were lysed by the Hirt procedure, and low-molecular-weight DNA in the supernatant was immunoprecipitated with anti-BrdU antibodies. The oligonucleotides were mixed with a 50-mer internal control, 3′ end-labeled, and analyzed on a DNA-sequencing gel. EdU excision product is produced by the wild-type NHF1 cells but not by the excision repair defective *XPA*^−/−^*XPC*^−/−^*CSB*^−/−^ cell line. (*E*) In vivo excision assay with TFIIH antibody immunoprecipitation. HeLa cells were treated as in (*C*), and the primary excision products were isolated by TFIIH immunoprecipitation. Purified primary products were mixed with a 50-mer internal control oligonucleotide, 3′ end-radiolabeled, and separated on a DNA-sequencing gel along with a DNA ladder. The excised oligomers are 25 to 32 nt in length, as is the case in excision repair of bulky adducts. Low-molecular-weight excised oligomers are not observed in TFIIH IPs, because after the excision products are released from TFIIH, they are degraded, and as seen in (*C*) and (*D*), can be immunoprecipitated with anti-BrdU antibodies.

Thymidine isotopes (^3^H-thymidine, or ^14^C-thymidine, or ^15^N-thymidine) were commonly used to label DNA in the 1950s because they are incorporated into DNA but not RNA (1–4). The time-consuming and low-resolution autoradiographic and density gradient separation assays used to detect the incorporation of these isotopes in DNA led to the development of halogenated derivatives of thymidine as an alternative tool to study DNA synthesis. The chemical structures of these thymidine analogs are very similar to thymidine (Fig. 1*A*). The methyl group at the 5 position of the thymine ring is replaced by halogen atoms (bromine [5-bromo-2′-deoxyuridine, BrdU], chlorine [5-chloro-2′-deoxyuridine, CldU], and iodine [5-iodo-2′-deoxyuridine, IdU]), and detection and visualization of these halogenated thymidine analogs is based mainly on immunochemical and immunofluorescence detection with anti-BrdU antibodies which cross react with CldU and IdU. One drawback of this immune-detection method is that it requires DNA denaturation with harsh chemical reagents to allow antibody accessibility of the halogenated thymidine derivatives in double stranded DNA which leads to instability of DNA and disruption of other cellular components.

In 2008, a strategy using 5-ethynyl-2′-deoxyuridine (EdU) and Cu-catalyzed azide alkyne cycloaddition (CuAAC) reaction (click chemistry) was developed to detect replicating DNA (5). EdU has a terminal alkyne group in the 5 position (Fig. 1*A*), which can be coupled with fluorescent- or biotin-labeled azides for fast, sensitive, and high-throughput detection without DNA denaturation. Since 2008, EdU has been widely used in the analysis of DNA replication (5–9), cell proliferation and differentiation (10), DNA combing (DNA fiber analysis), and measuring nucleotide excision repair synthesis in the form of “unscheduled DNA synthesis” (11–14). Despite its utility and widespread use, EdU is actually highly toxic (15, 16), causing more cell death than the other thymidine analogs used to study replication, and the underlying molecular mechanism of toxicity is not known.

Excision repair recognizes and removes bulky DNA lesions induced by a variety of DNA-damaging agents, including environmental carcinogens such as UV radiation (17), benzo(*a*)pyrene (BaP) (18) from cigarette smoke, and anticancer drugs such as cisplatin (19, 20). Here, we show that the widely used thymidine analog EdU is actually a substrate for excision repair, unlike the chemically related analogs BrdU, CldU, IdU, (2′S)-2-deoxy-2-fluoro-5-ethynyluridine (F-ara-EdU), or 5-(azidomethyl)-2′-deoxyuridine (AmdU). We discovered EdU excision unexpectedly while screening these thymidine analogs to investigate nucleotide excision repair patch boundaries. As is the case with DNA containing other “conventional” damage such as photoproducts induced by UV radiation, DNA containing EdU is removed in the form of 22 to 30 long oligomers (nominal 26-mer) by the excision nuclease. Since the excised oligomers remain in a tight complex with the repair factor TFIIH, we were able to map EdU repair in the human genome at single-nucleotide resolution by performing the excision repair sequencing (XR-seq) method (17). We observed that EdU was excised throughout the genome and was subject to transcription-coupled repair (TCR) as evidenced by higher repair rates in the transcribed strand (TS) relative to the nontranscribed strand (NTS) in transcriptionally active genes. These properties of EdU, combined with its ability to cross the blood–brain barrier, make it a potential candidate for the treatment of brain cancers, in particular, glioblastomas (21–23).

## Results

### Incorporation of Thymidine Analogs into the Human Genome and Removal of EdU by Nucleotide Excision Repair.

UV-irradiated human cells excise cyclobutane pyrimidine dimers (CPDs) and (6, 4) photoproducts ((6, 4) PPs) in the form of nominal 26 nucleotide-long oligomers (24–26), which may be purified by damage- or TFIIH-specific antibodies to be analyzed directly, or sequenced and mapped to the genome by the XR-seq method (17, 19, 20, 26, 27). In addition to using damage-specific or TFIIH-specific antibodies to capture the excised oligomers, we wanted to use antibodies to readily available pyrimidine analogs (Fig. 1*A*). EdU, BrdU, CldU, and IdU can be immunoprecipitated with anti-BrdU antibodies (28, 29) and are similarly incorporated into replicating HeLa cells (Fig. 1*B*).

Before using the anti-BrdU antibodies to capture the excised oligomers from UV-irradiated cells, we proceeded to perform a series of experiments as a negative control of UV treatment that gave some unexpected results. HeLa cells replicating in the presence of either EdU, BrdU, CldU, or IdU were collected, lysed, and supernatants were immunoprecipitated with anti-BrdU antibodies. The eluted material was mixed with an internal standard control 50-mer, radiolabeled, and separated on a sequencing gel. Surprisingly, the BrdU antibodies precipitated oligomers in the form of nominal 26-mer from EdU-labeled cells, but not cells labeled with BrdU, CldU, or IdU (Fig. 1*C*). This raised the possibility that EdU in the genome was recognized as damage and processed by nucleotide excision repair as such.

### Repair-Deficient Cells Do Not Excise EdU, and Other Thymidine Analogs Are Not Excision Repair Substrates.

To confirm that EdU-containing DNA is a bona fide nucleotide excision repair substrate, we assayed repair using an *XPA*^−/−^ *XPC*^−/−^ *CSB*^−/−^ derivative of NHF1 cells, which lack the capacity to perform either global or TCR. Fig. 1*D* shows that the mutant has a clean background and no excision product, in contrast to the excision product seen with the wild-type NHF-1 parental cell line. Thus, taken together, the data lead us to conclude that EdU is removed from the genome by nucleotide excision repair.

Having found that EdU is a substrate for the human excision nuclease, we proceeded to screen the other two thymidine analogs (F-ara-EdU and AmdU) that are used to monitor cell proliferation and DNA replication dynamics but are not recognized by anti-BrdU antibodies by isolating the potential excision products containing the analogs using TFIIH antibodies. We have previously shown that excised oligonucleotides remain in a tight complex with TFIIH, which also protect them from nonspecific nucleolytic degradation following release from the genomic DNA (17, 30, 31). Fig. 1*E* shows that with anti-TFIIH antibodies, only EdU gives rise to the 25- to 32-nt-long excision products, and as noted, under these conditions, only the undegraded primary excision product is detected, in contrast to the BrdU immunoprecipitates (IPs), in which the smaller degraded excision products are also seen (Fig. 1*C* and *D*). Thus, we conclude that of all of the thymidine analogs currently in use for studying DNA replication, only EdU is excised by human nucleotide excision repair.

### Capturing Excised Oligomers from UV-Irradiated Cells with Anti-BrdU Antibodies.

**Fig. 2.**
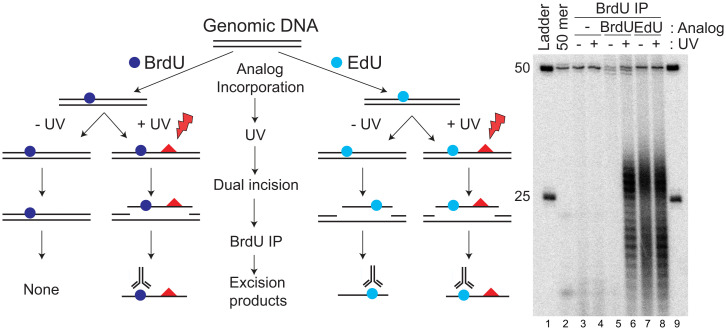
EdU is a substrate for excision repair. Anti-BrdU antibody precipitates excision products from EdU-labeled, unirradiated cells. HeLa cells were either not treated with thymidine analog or treated with 10 μM EdU or BrdU for 24 h, then irradiated with UV (20 J/m^2^) where indicated. Red triangles indicate CPDs produced by UV exposure. After 1 h of repair in the presence of analog, excised oligonucleotides were separated from cells, immunoprecipitated with anti-BrdU antibodies, and then radiolabeled at the 3′ end using α-[^32^P]ATP and TdT, and analyzed on a sequencing gel.

As our original goal was to use the anti-BrdU antibodies to capture the excised oligomers from UV-irradiated cells, we incubated HeLa cells in medium containing either BrdU (24, 32, 33) or EdU (5, 34) for 24 h before UV irradiation, to permit incorporation of the analogs into the genome, and thus, the excision products. Then, 1 h after UV irradiation (20 J/m^2^, 254 nm) cells were collected and lysed and small oligonucleotides were immunoprecipitated, mixed with an internal standard control 50-mer, radiolabeled, and separated on a sequencing gel. UV excision products were detected in cells incubated with either BrdU or with EdU, but not in the cells without analog (Fig. 2). A similar amount of EdU-containing oligos are excised in the presence and absence of UV, which indicates that EdU is a good substrate for nucleotide excision repair.

### Repair of EdU by Mammalian Cell-Free Extract (CFE).

**Fig. 3.**
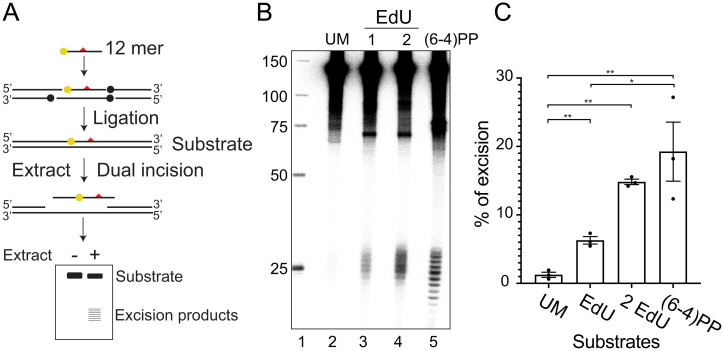
EdU-substituted DNA is recognized as damage and excised by nucleotide excision repair in vitro. (*A*) Schematic of substrate synthesis and in vitro excision of EdU by mammalian CFE. 140 bp duplex substrates were synthesized with a ^32^P label adjacent to a uniquely located modification. Modifications (red triangles) included a (6, 4) PP, an EdU, or two EdUs. Substrates were synthesized by phosphorylating, annealing, and ligating six component oligonucleotides. Ligation sites are indicated by dots. The central oligonucleotide containing the modification was labeled at the 5′ end with ^32^P (yellow dot site). A control UM substrate was also synthesized (not shown). Substrates were incubated with CFE, and unreacted full-length substrate and excised products were purified and resolved with a sequencing gel. (*B*) Result of a representative experiment. (*C*) Percentage of excision products relative to substrate was quantified based on three biological replicates. Data are means ± SEMs; *n* = 3. *P < 0.05, **P < 0.01, two-tailed unpaired *t* test.

To further confirm that EdU is a bona fide substrate for excision repair and to compare its excision relative to a (6, 4) photoproduct, we conducted an in vitro assay with mammalian CFEs using duplex DNA substrates (Fig. 3*A*) that are either unmodified (UM) or possess one or two EdU residues or a (6, 4) photoproduct adjacent to a ^32^P radiolabel (35). As is evident in Fig. 3*B*, both EdU substrates are excised with products in the form of a nominal 26-mer (22 to 30 nt in length) comparable to the (6, 4) photoproduct. Quantification of the percentage of excision relative to the total amount of substrate based on three biological replicates (Fig. 3*C*) shows that the substrate containing one EdU was excised less efficiently compared with the (6, 4) PP. The substrate with two EdUs gave a stronger signal than the substrate with one EdU, which indicates that two EdUs may cause more severe DNA helical distortions than one EdU. Thus, the in vitro excision assay confirms that EdU is an efficient substrate for the mammalian excision nucleases.

### Light-Independent EdU Excision.

Since EdU has a high extinction coefficient in the 280- to 300-nm region (see *SI Appendix*, Fig. S1*A*), we considered the possibility that cells labeled with EdU exposed to the light in the room during routine manipulation may have formed an EdU adduct with another cellular component generating a “bulky ” adduct recognizable by the excision nuclease. To address that possibility, we did a carefully controlled experiment in which one set of the assays were performed under yellow light and one set under ordinary room light (see *SI Appendix*, Fig. S1*B*). Under both conditions, cells grown in EdU-containing media excised oligonucleotides 22 to 30 nt in length. Thus, we conclude that the excision is not caused by an EdU photoproduct, but by EdU itself.

### DNA-EdU Substrate Formation and Decay Kinetics.

**Fig. 4.**
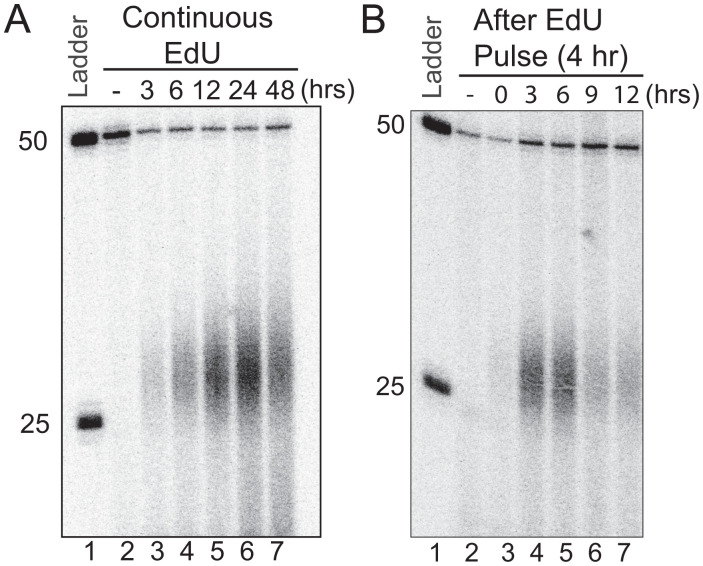
Decay kinetics of the EdU-induced excision products in human cells. (*A*) In vivo excision assay showing EdU excision dynamics. HeLa cells were treated with 10 μM EdU, for 3, 6, 12, 24, and 48 h. Then, the excised oligonucleotides were isolated, immunoprecipitated with anti-TFIIH antibodies, mixed with a 50-mer internal control, 3′ end labeled, and separated on DNA-sequencing gels. (*B*) In vivo excision assay of EdU pulse labeling and decay kinetics. HeLa cells were pulse labeled for 4 h in medium containing 10 μM EdU. Then, EdU-containing medium was removed and cells were supplied with fresh medium and incubated at 37 °C for 0, 3, 6, 9, and 12 h. Then, the excised oligonucleotides were isolated, immunoprecipitated with anti-TFIIH IP antibodies, and analyzed as in (A).

To determine the optimal time for EdU substrate formation in the genome, we incubated HeLa cells in medium containing 10 μM EdU for 3 to 48 h, then lysed cells, isolated excision products using BrdU antibodies, and detected the excised EdU-containing fragments by 3′ labeling and autoradiography. Fig. 4*A* shows that the peak level is reached at 24 h and declines by 48 h. However, the 48-h decline is due to the loss of total genomic DNA caused by cell death, potentially the consequence of the long EdU exposure and continuous excision and resynthesis. To determine the kinetics of excision after the initial incorporation of EdU, we incubated cells with EdU for 4 h, then replaced the medium with EdU-free medium and followed EdU excision at time points up to 12 h. As seen in Fig. 4*B*, removal continues for at least 12 h, albeit with a gradual decrease in the excision products.

### EdU in the Human Genome Is Subject to TCR.

**Fig. 5.**
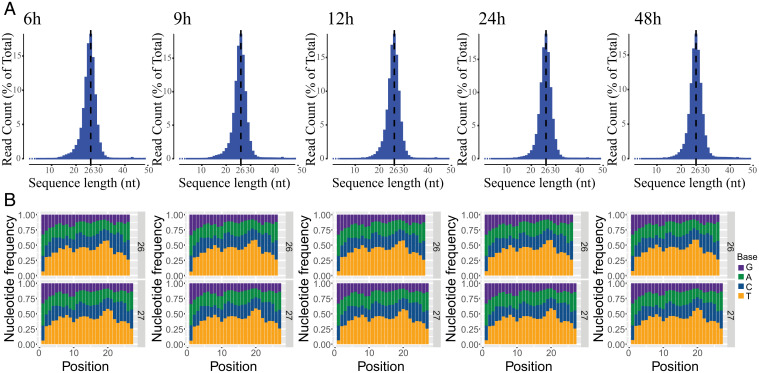
XR-seq analysis: Frequency distribution profiles of excision product length and nucleotide composition following different EdU treatment times. (*A*) The size distribution of excision products peaks at 25 to 27 nt. (*B*) Nucleotide distribution of the 26-mer and 27-mer excision products. Excision products generated by repair typically have the damaged bases located at positions 19 to 21; consistent with this is the peak in T residues (potential sites of EdU substitution) seen here surrounding position 20. Position is the distance in nucleotides from the 5′ end.

**Fig. 6.**
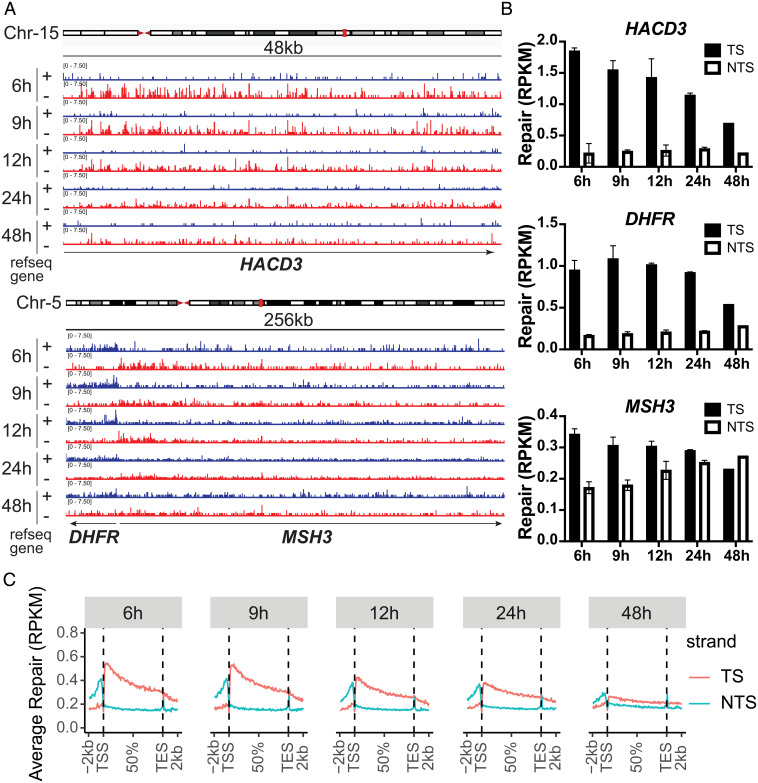
EdU in the human genome is subject to transcription coupled repair. (*A*) Browser view of representative genes clearly show transcription-coupled repair. EdU repair on the *HACD3*, *DHFR*, and *MSH3* genes. “+” indicates plus-strand DNA (blue), “−” represents minus-strand DNA (red). Minus strand is the TS for *HACD3* and *MSH3*, and the plus strand is the TS for *DHFR*. Individual bars represent the number of excision product reads as reads per kilobase per 10 million total reads on a scale from 0 to 7.5. Average repair of each gene was quantified in the bar chart in (*B*). (*C*) Genome-wide EdU repair profile across all genes on the TS and NTS at 6, 9, 12, 24, and 48 h treatment times. In (*B*) and (*C*), RPKM is reads per kilobase per million total reads. Data for each strand were scaled to a unit gene to represent average repair in RNA Pol II-transcribed genes and the 2 kb upstream and downstream.

Structurally, the ethynyl group at the C-5 position in uridine in EdU is not drastically different from the –CH_3_ group at this position of thymidine. Hence, it was not clear whether EdU would arrest DNA or RNA polymerases, which in the latter case is assumed to be important for DNA adduct removal by TCR. We tested EdU as a potential polymerase block by performing PCR: using Taq DNA polymerase and genomic DNAs from untreated cells or cells treated with EdU for 24 h, we found that under both conditions, the PCR produces full-size fragments (see *SI Appendix*, Fig. S2). With this background, we could not predict whether EdU would block RNA polymerase II (RNA Pol II), and thus be subjected to TCR. To find out, we performed XR-seq on excision products that were immunoprecipitated with anti-TFIIH antibodies and thus presumed to be the primary reaction products. Fig. 5*A* shows the size distribution of the excision products, which exhibits the expected narrow length distribution with a peak at 25 to 27 nt. The nucleotide distribution of the excision products in this range shows enrichment of TS at positions 19 and 20 for 26-mers and at positions 20 to 21 for the 27-mer (Fig. 5*B*). Browser views of the HACD3, DHFR, and MSH3 genes that have been extensively used for studying TCR of UV damage (17) clearly show TCR (Fig. 6*A*). The TS:NTS repair ratio peaks at 6 h and gradually decreases from 9 to 48 h (Fig. 6*B*).

The plots in Fig. 6*C* show analysis of TS versus NTS repair genome-wide. XR-seq reads from nonoverlapping genes known to be transcribed by RNA Pol II were scaled to a unit gene, and reads 2 kb upstream and downstream of each gene were included in the plots. The TS:NTS ratio is ∼2.5 at 6 h and decreased to ∼1.2 at 48 h, with higher values in promoter-proximal than distal regions, and there is a switch of preferential repair upstream of the transcription start site known to be caused by antisense transcription in the promoter and enhancer regions of RNA Pol II transcribed genes (27).

### Effect of Chromatin State on EdU Repair.

Open or closed chromatin affects the accessibility of repair proteins and further affects repair efficiency. A total of 15 chromatin states were predicted by the ChromHMM algorithm based on chromatin modifications (36). Our previous reports showed that chromatin states affect the repair efficiency of CPD, (6, 4) PP (37), cisplatin (38), and 10-(deoxyguanosin-N^2^-yl)-7,8,9-trihydroxy-7,8,9,10-tetrahydrobenzo(*a*)pyrene (BPDE-dG) adducts (18). We wished to test whether the same also applied to EdU in the genome. Using a previously described method (37), we calculated the number of XR-seq reads in each chromatin state and found that the reads are relatively high over the active promoter, weak promoter, strong enhancer, and transcription transition region (see *SI Appendix*, Fig. S3*A*), and these reads decrease over time (6 to 48 h). This enrichment of reads at active chromatin regions is additional evidence that EdU repair is transcription coupled.

Next we mapped the EdU XR-seq reads over the DNase hypersensitivity peaks of gene and intergenic regions. Repair of EdU was significantly enriched at all DNA hypersensitivity peaks and the repair at gene regions was more efficient than intergenic regions because it is more accessible by repair proteins, and repair of EdU in gene regions gradually decreased with time (see *SI Appendix*, Fig. S3*B*). Repair of EdU around the DNase hypersensitivity peaks of intergenic regions increased with time as the repair of gene regions decreased.

## Discussion

Excision repair removes a wide range of dissimilar lesions from the genome by dual incision. Nucleotide excision repair acts on bulky adducts such as UV-induced CPD, (6, 4) PP, and cisplatin adducts, but also removes lesions that cause minor helical distortions compared with bulky adducts (35, 39), including phosphorothioate (S), methylphosphonate-modified thymidine, thymidine glycol, and 8-oxoguanine (8-oxoG). Theoretically, all thymidine analogs that are modified in the 5 position of the thymidine ring could be substrates of excision repair; however, only EdU is excised in sufficient quantities to be detectable by our assays. The reason for this is unclear. Initially, we considered that EdU may induce interstrand crosslinks and form bulky adducts that are subject to excision repair because the alkyne group (triple bond) is highly reactive. However, our experiments revealed no evidence for excision resulting from EdU reactivity. Notably, we found that in XR-seq, EdU can be amplified by PCR without a damage reversal step (see *SI Appendix*, Fig. S2), since the DNA polymerase can bypass EdU adducts. Thus, it is highly unlikely that the EdU excision we detect is caused by a bulky adduct formed by EdU reacting with other cellular components. This is supported by results obtained with the in vitro assay (Fig. 3) using defined single EdU or double EdU DNA substrates in which no interstrand crosslinks were introduced. Both single and double EdU substrates were recognized and excised by mammalian excision nuclease. We concluded that thymidine base modifications, but not interstrand crosslinks of EdU, contribute to its excision. The differences between thymidine analogs in terms of helical distortion caused by substitution of the –CH3 group with different atoms remain to be investigated.

EdU, like other thymidine analogs, crosses the blood–brain barrier and has been used to examine the limited DNA replication in brain cells (5, 40, 41). However, studies of replication in the brain are limited because cell division in the brain is confined largely to the developmental phase. In contrast, cancers are characteristically composed of dividing cells. It has been previously shown that EdU outperforms BrdU in suppressing cell proliferation in an osteosarcoma and a glioblastoma cell line (21). Perhaps more important, even though both EdU and temozolomide (the standard of care in treating glioblastoma) suppressed primary human glioblastoma cell proliferation to similar levels, EdU induced three- to fivefold more strand breaks and apoptosis than temozolomide at the same dose (21). Our discovery of an EdU-initiated futile repair reaction may explain these findings that have remained unexplained and raises the possibility of its use in glioblastoma treatment in model systems and eventually in a clinical setting.

## Materials and Methods

### Cell Lines.

NHF1 cells, telomerase-immortalized normal human fibroblast monolayers, were obtained as described previously (17), *XPA*^−/−^*XPC*^−/−^*CSB*^−/−^ triple knockout NHF1 cells were generated using the CRISPR-Cas9 system with guide RNAs (gRNAs) (*XPA*: GGCGGCTTTAGAGCAACCCG; *CSB*: CGTGGAGAAGGAGTATCGGT; *XPC*: CGAGATGTGGACACCTACTA). The HeLa cell line came from the American Type Culture Collection. NHF1 and HeLa cells were maintained in Dulbecco’s modified Eagle’s medium supplemented with 10% fetal bovine serum at 37 °C in a 5% CO_2_ humidified chamber.

### Reagents and Antibodies.

Thymidine analogs EdU (900584), BrdU (B5002), IdU (I7125), CldU (C6891), F-ara-EdU (T511293), and AmdU (T342254) were purchased from Sigma. Anti-BrdU antibody (Bu20A) was from Thermo Fisher (14-5071-82); anti-p89 (sc-293), and anti-p62 (sc-292) were purchased from Santa Cruz Biotechnology; and anti-single-stranded DNA (ssDNA) antibody was from Millipore Sigma (MAB3034). Undamaged (AGGAATTAAGGA), single EdU (AGGAATEdUAAGGA), and double EdU (AGGAAEdUEdUAAGGA) modified oligomers were synthesized by Integrated DNA Technologies (IDT).

### UV Irradiation and Thymidine Analog Treatment.

HeLa cells were grown to ∼80% confluence, culture medium was removed, cells were washed twice with phosphate-buffered saline (PBS), and then were placed under a GE germicidal lamp emitting primarily 254-nm UV light (1 J/m^2^/s) connected with a digital timer for 20 s (20 J/m^2^ in total). Cells were collected after 1 h of repair. Thymidine analogs were dissolved in sterile PBS (BrdU, EdU, CldU) or dimethylsulfoxide (IdU, F-ara-EdU, AmdU), and then added to cell culture medium at a final concentration of 10 μM for the indicated times.

### Slot Blot Assay.

HeLa cells were plated in 100-mm plates and treated with 10 μM thymidine analogs for 24 h. Genomic DNA was purified from cell pellets using the QIAamp DNA mini kit (Qiagen, 51306). DNA concentrations were determined using a Qubit 3.0 fluorometer. A total of 250 ng of each DNA was diluted with Tris-EDTA buffer (10 mM Tris⋅HCl, pH 8, 1 mM EDTA) to a total volume of 250 μL, then boiled at 90 °C for 10 min and cooled in ice water immediately. A total of 250 μL cold ammonium acetate (2 M) was added to neutralize DNA, and then DNA samples were loaded onto nitrocellulose membranes using a slot blot apparatus. Membranes were transferred to a vacuum oven and baked for 1.5 h at 80 °C, then blocked with 5% dried milk and incubated with either anti-BrdU antibody (1:1,000) or anti-ssDNA antibody (1:5,000) as loading control overnight at 4 °C. After incubating with a horseradish peroxidase–labeled secondary antibody (1:10,000) for 1 h, chemiluminescent signal was captured using a Bio-Rad ChemiDoc XRS imaging device.

### In Vivo Excision Assay.

For the in vivo excision assay with BrdU antibody, low-molecular-weight DNAs isolated by the Hirt procedure as previously described (17) were subject to immunoprecipitation with anti-BrdU antibody. For the in vivo excision assay with TFIIH antibody, cells were lysed in buffer A (25 mM HEPES at pH 7.9, 100 mM KCl, 12 mM MgCl_2_, 0.5 mM EDTA, 2 mM dithiothreitol (DTT), 12.5% glycerol, and 0.5% Nonidet P-40), and excised oligonucleotides complexed with TFIIH protein were immunoprecipitated with TFIIH antibody. Purified oligonucleotides from BrdU IP or TFIIH IP were 3′ end-labeled by terminal deoxynucleotidyl transferase (TdT), α-[^32^P]ATP (adenosine triphosphate) with a standard 50-mer oligonucleotide as spike internal control. After phenol-chloroform extraction and ethanol precipitation, labeled DNAs were fractionated with 12% denaturing sequencing gels.

### In Vitro Excision Assay.

Radiolabeled 140-bp full-length undamaged, (6, 4) PP, and EdU-damaged substrates were prepared, and in vitro excision assay with CFE from Chinese hamster ovary (CHO) AA8 cells were carried out as previously described (39). Briefly, CHO AA8 CFE was prepared using the method of Manley (42). Linear double-stranded DNA substrates (140 bp in length) were prepared with centrally located (6, 4) PP or EdU as described previously (43). The sequence of the centrally located 12-mers was 5′-AGGAATTAAGGA. For the (6, 4) substrate, the (6, 4) lesion was between T5 and T6. Single EdU was at T6 and double EdU lesions were at T5 and T6. Unmodified 12-mer and EdU containing 12-mers were purchased from IDT.

The excision reaction with AA8 CFE (75 µg) was conducted with 20 fmol of 140-bp substrates, 18 mM HEPES-KOH (pH 7.9), 24 mM KCl, 2 mM MgCl_2_, and 4 mM ATP at 30 °C for 60 min as described (35). After the excision reaction, the mixture was incubated with 0.34% sodium dodecyl sulfate and 20 µg/mL proteinase K at 55 °C to 60 °C for 15 min. Then, DNA was extracted with phenol:chloroform:isoamyl alcohol and precipitated with ethanol, resuspended in formamide/dye mixture, and separated with a 10% sequencing gel. Quantification of the signal intensities was done by using ImageJ.

### XR-Seq.

The XR-seq experiment was done as previously reported (17, 27), with modifications. In brief, HeLa cells were treated with 10 μM EdU and harvested after 6, 12, and 24 h. Cell pellets from two 150-mm culture plates were suspended and lysed in 1 mL cold buffer A (25 mM HEPES at pH 7.9, 100 mM KCl, 12 mM MgCl_2_, 0.5 mM EDTA, 2 mM DTT, 12.5% glycerol, and 0.5% Nonidet P-40). The primary EdU excision products were isolated from the soluble fraction by IP with TFIIH antibody followed by ligation of 5′ and 3′ adapters. After adaptor ligation, excised oligomers containing EdU were further purified by IP with anti-BrdU antibody that also reacts with EdU. Purified EdU excision oligomers were directly subjected to PCR amplification using 50- and 63-nt-long primers that introduce specific barcodes compatible with the Illumina TruSeq small RNA kit, without a damage reversal step as is done with CPD or (6, 4) PPs. The PCR products containing excised oligonucleotides were ∼145 bp in length and resolved with a 10% nondenaturing gel. EdU excision oligomers from different time points were gel purified, pooled, and sequenced on the NextSeq-P3 platform at the University of North Carolina–Chapel Hill High-Throughput Sequencing Facility.

### Data Analysis.

Analysis of sequencing reads and data visualization was as described previously (19). Reads were trimmed to remove flanking adapter sequences by cutadapt (44), and then duplicate reads were removed by fastx_toolkit/0.0.14 (hannonlab.cshl.edu/fastx_toolkit/index.html). Trimmed reads were aligned to hg38_UCSC by using bowtie2 with arguments -f -very-sensitive (45, 46). The output .sam files were converted into .bam files by using SAMtools (47) and then were converted into .bed files using bedtools (48). Oligonucleotide lengths and nucleotide distributions were plotted by R. Only the reads of 26-mer length with T at 19 and 27-mer length with T at 20 were analyzed.

Bigwig files were visualized by IGV (RRID: SCR_007073, Broad Institute, and the Regents of the University of California) (49, 50). Reads per kilobase per million mapped reads (RPKM) for each gene were plotted with Prism 9 (RRID: SCR_ 002798). For plotting average repair profiles as a unit gene, we chose genes with length >5 kbp, and the distance between genes was at least 5 kbp. For chromatin state analysis, bedtools (48) coverage was used to calculate the repair levels over each of the 15 predicted chromatin states defined by the ChromHMM algorithm (36). Values were normalized per million mapped reads and per kilobase of interval length and plotted with R (https://github.com/yanyanyangunc/DNA-Damage-Repair-Circadian-Clock/tree/master/excisionRepair). DNase-seq (accession no. ENCSR000EMP) fastq, aligned reads .bam files, and peak files, as well as the NHLF chromHMMchromatin state segmentation (UCSC accession no. wgEncodeEH000792), were downloaded from the ENCODE portal (genome.ucsc.edu/ENCODE/).

The raw data and alignment data have been deposited in the Gene Expression Omnibus under accession number GSE202784.

## Supplementary Material

Supplementary File

## Data Availability

High-throughput sequencing data have been deposited in Gene Expression Omnibus GSE202784(51). All of the study data are included in the article and/or supporting information.
