# Supplementary material for: Nucleotide excision repair removes thymidine analog 5-ethynyl-2′-deoxyuridine from the mammalian genome
Source: Proc Natl Acad Sci U S A. 2022 Aug 22;119(35):e2210176119. doi: 10.1073/pnas.2210176119 (PMC9436350; doi:10.1073/pnas.2210176119)
Supplement: Supplementary File [file pnas.2210176119.sapp.pdf]

Supplementary Information for

Nucleotide Excision Repair Removes Thymidine Analog 5-Ethynyl-2'-deoxyuridine From the Mammalian Genome

Li Wang[1+], Xuemei Cao[1+], Yanyan Yang[1+], Cansu Kose[1], Hiroaki Kawara[1], Laura A. Lindsey-Boltz[1], Christopher P. Selby[1], Aziz Sancar[1*]

Aziz Sancar
Email**:** aziz_sancar@med.unc.edu

This PDF file includes:

Figures S1 to S3

A

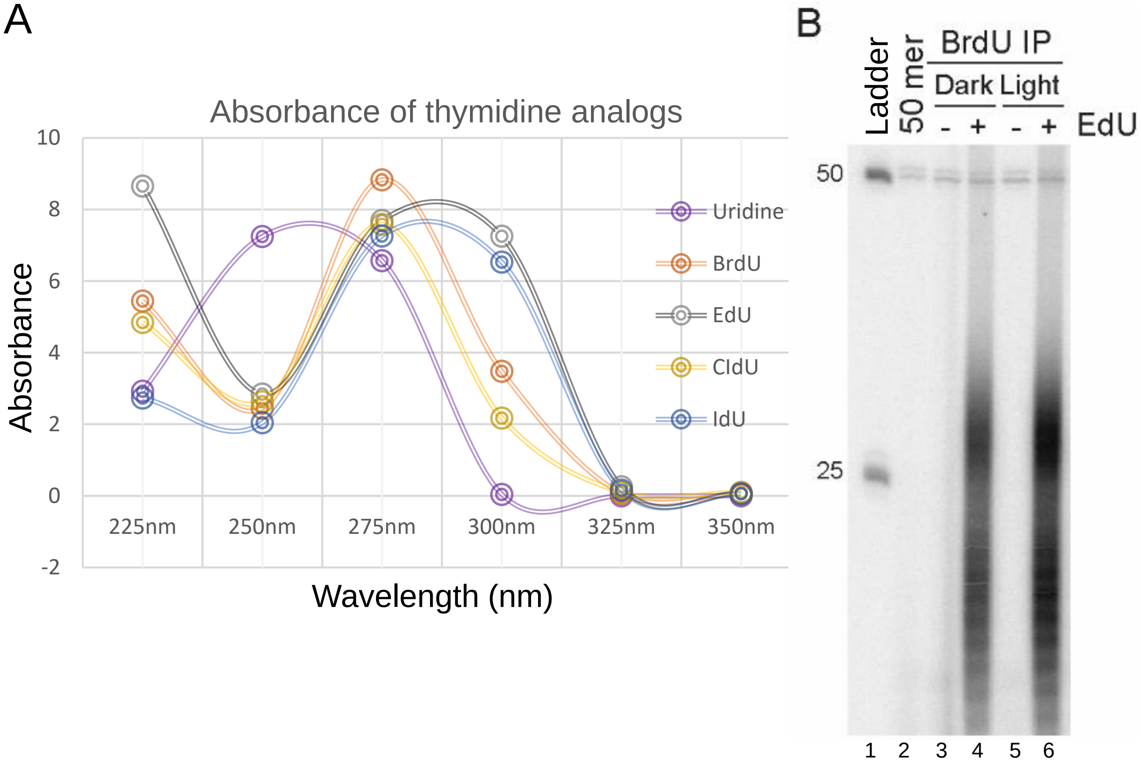

Fig. S1. Absorbance of thymidine analogs. (A) Thymidine analogs were diluted to 1 mM and absorbance of each was measured using a NanoDrop spectrophotometer. (B) The nucleotide excision is not caused by EdU photoproducts but by EdU itself. HeLa cells were grown in a dark chamber with or without 10 μM EdU for 24 hours. Samples were either processed in the dark (protected from light) or under regular light condition and analyzed on a sequencing gel.

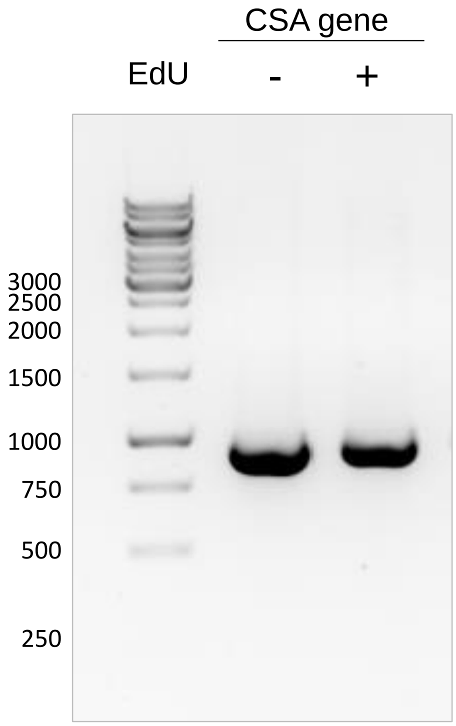

Fig. S2. Taq DNA polymerase can bypass EdU adducts. Genomic DNA from HeLa cells treated with or without 10 mM EdU for 24 h was isolated. 50ng of DNA was used as template for PCR amplification of the CSA gene (946bp) with Taq polymerase for 30 cycles. EdU substituted DNA template gave nearly as much full length PCR products as unsubstituted DNA.

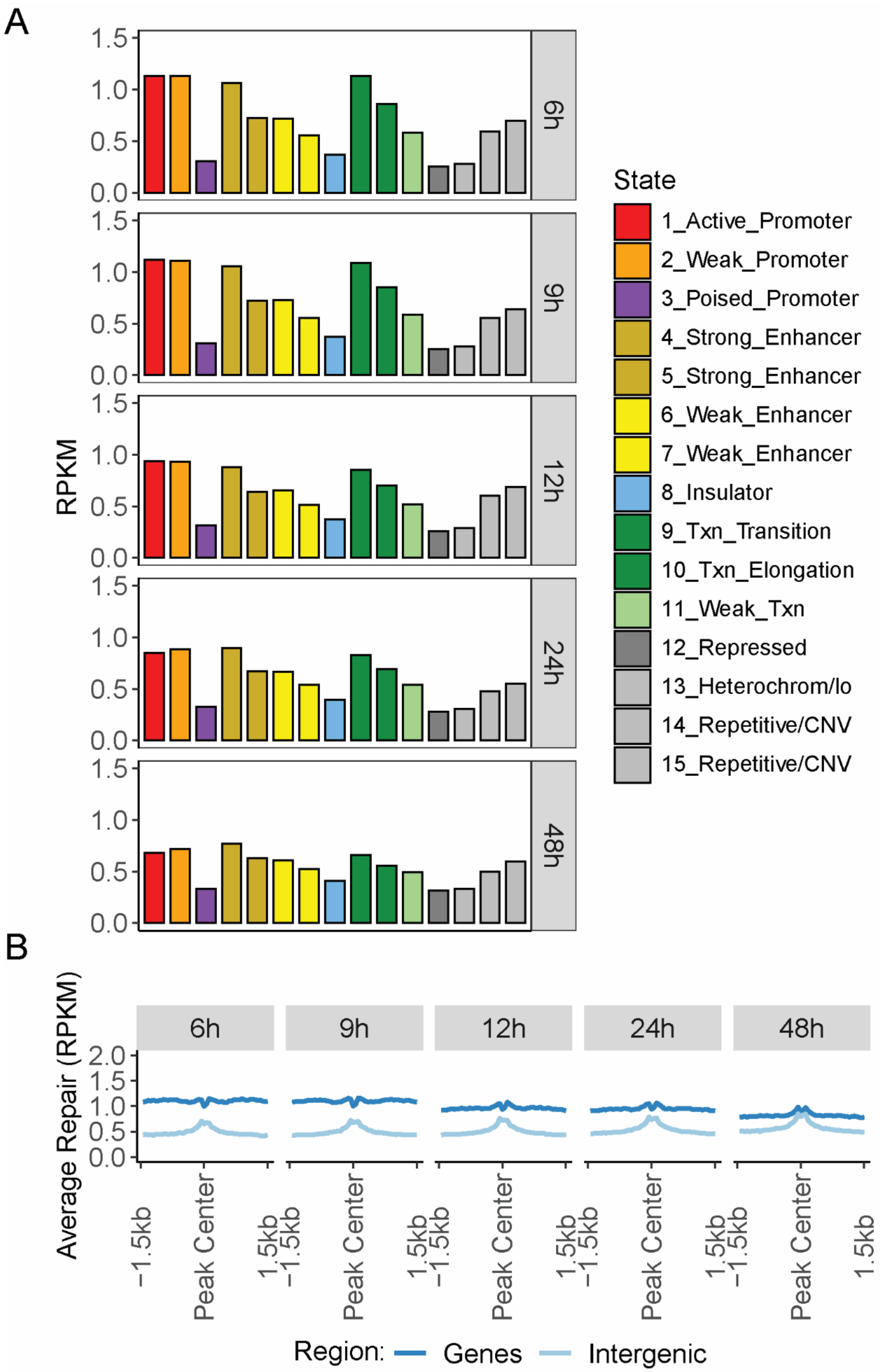

Fig. S3. Effect of chromatin state on EdU repair. (A) XR-seq read coverage was calculated over genomic intervals assigned to each of the chromatin states predicted for NHLF cells (ENCODE). Shown are results from EdU XR-seq at the different time points (6 h, 9 h, 12 h, 24 h and 48 h). Values were normalized to read depth and interval length. (B) Average EdU XR-seq profiles for each time point at 1.5-Kb regions flanking the center of DNase hypersensitivity peaks that either overlapped annotated genes (Genes) or did not overlap annotated genes (Intergenic).
